# Supplementary figures and images for: C5aR agonist enhances phagocytosis of fibrillar and non-fibrillar Aβ amyloid and preserves memory in a mouse model of familial Alzheimer’s disease
Source: PLoS One. 2019 Dec 6;14(12):e0225417. doi: 10.1371/journal.pone.0225417 (PMC6897413; doi:10.1371/journal.pone.0225417)

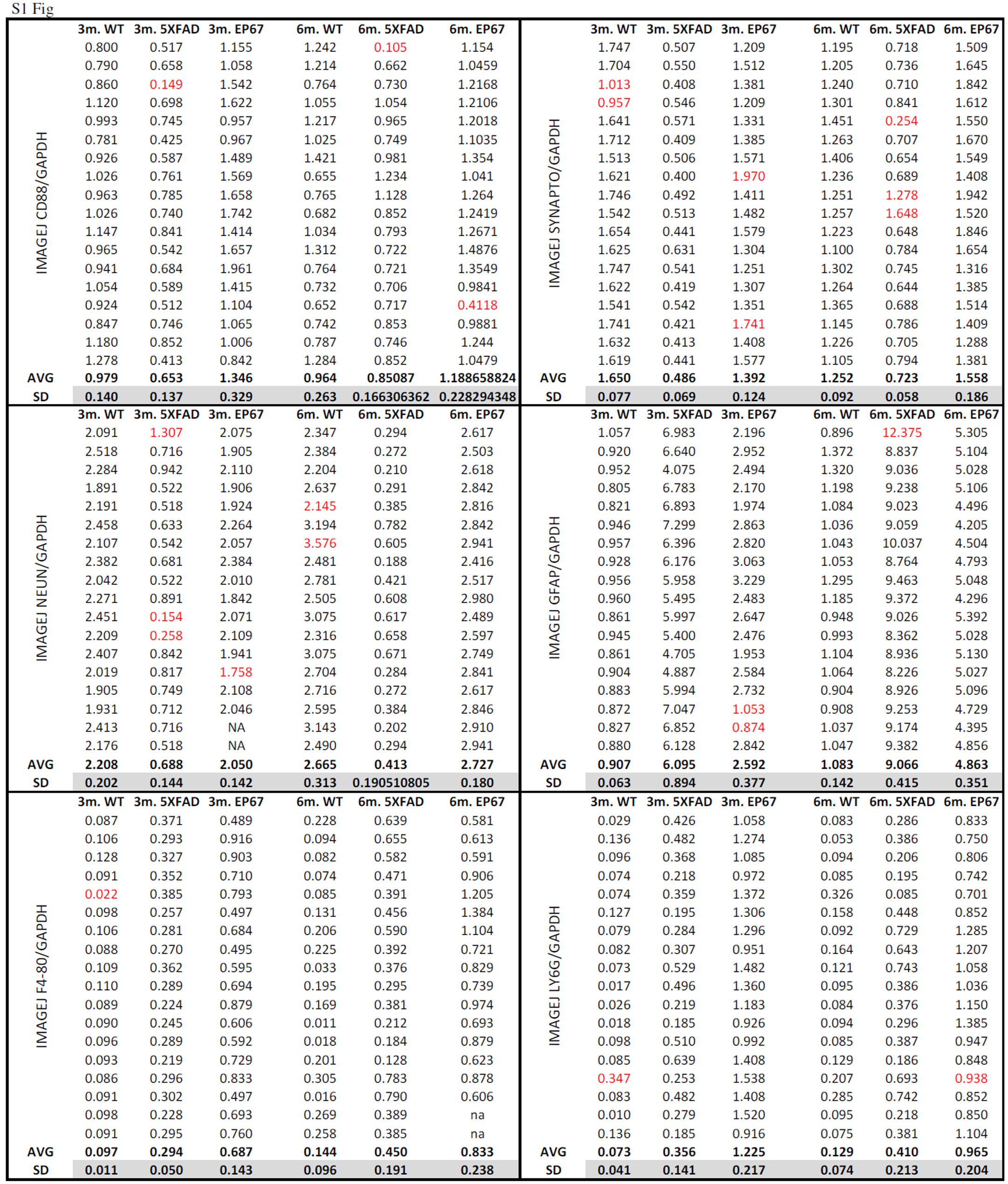

Supplement: S1 Fig — Raw data from immunoblot experiments. (TIF) [file pone.0225417.s001.tif]

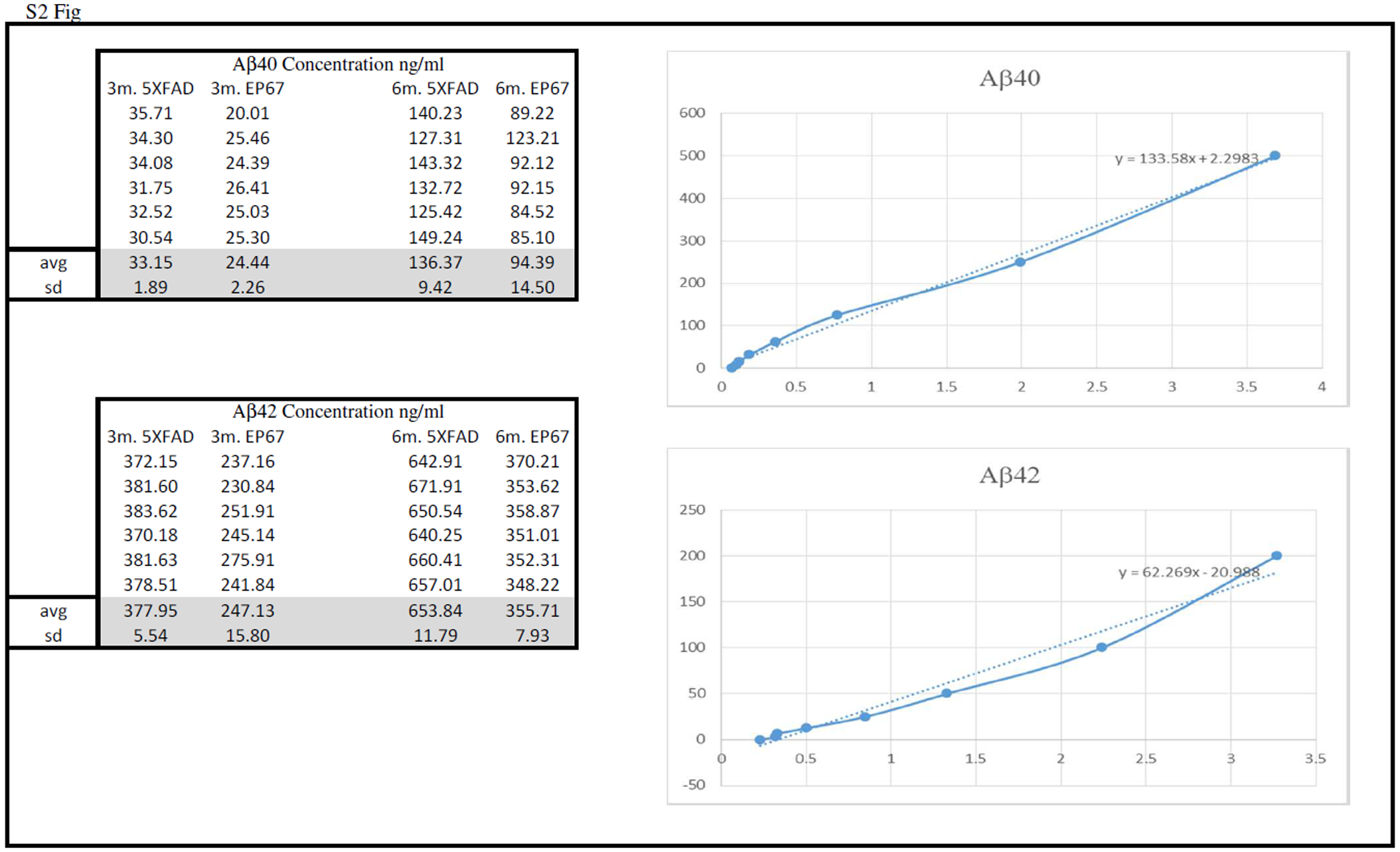

Supplement: S2 Fig — Raw data from Aβ42 and Aβ40 immunoassay kits. (TIF) [file pone.0225417.s002.tif]

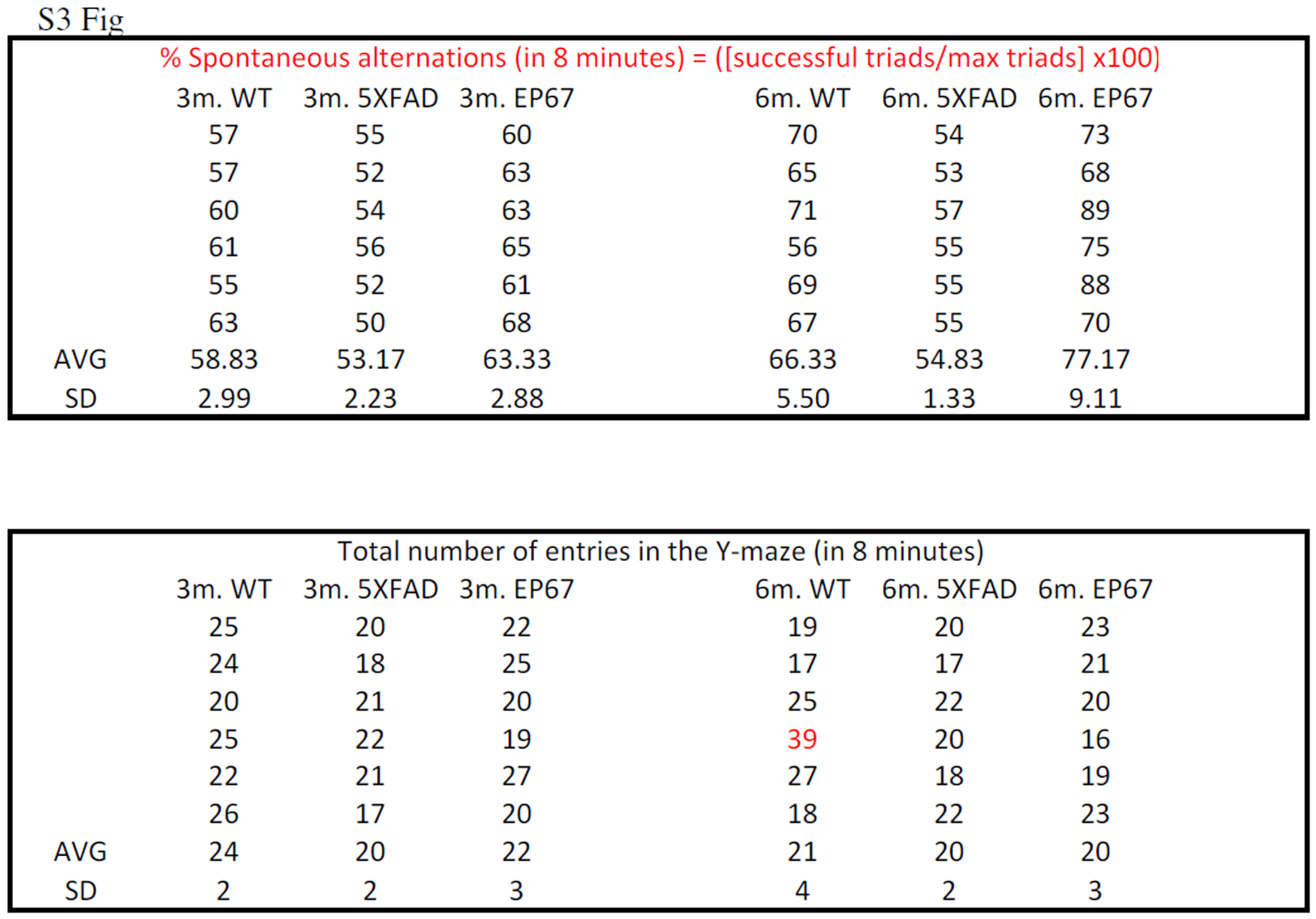

Supplement: S3 Fig — Raw data obtained during Y-maze spontaneous alternation test. (TIF) [file pone.0225417.s003.tif]

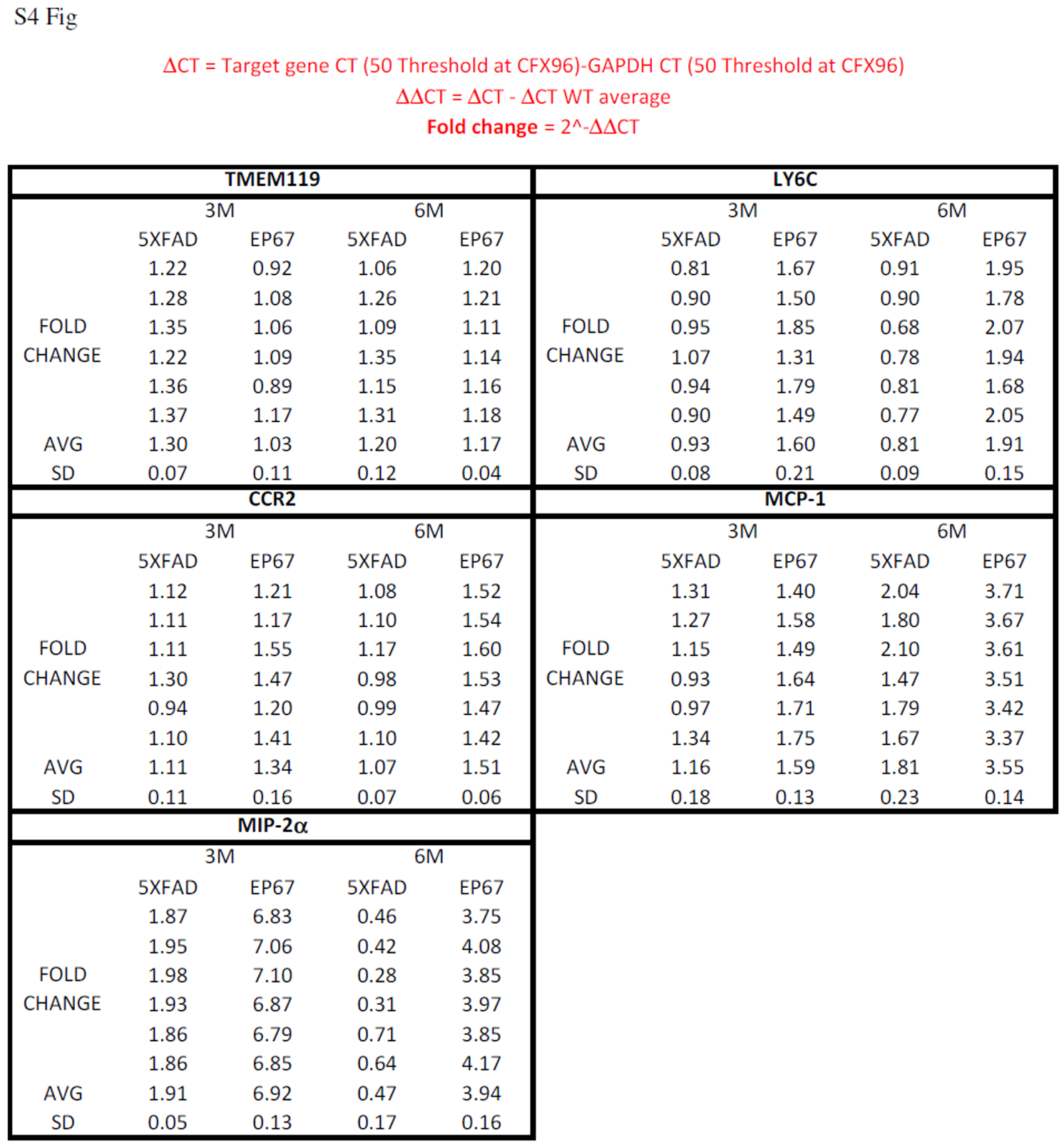

Supplement: S4 Fig — Raw data from the various real-time PCR targets. (TIF) [file pone.0225417.s004.tif]
