# Supplementary material for: C5aR agonist enhances phagocytosis of fibrillar and non-fibrillar Aβ amyloid and preserves memory in a mouse model of familial Alzheimer’s disease
Source: PLoS One. 2019 Dec 6;14(12):e0225417. doi: 10.1371/journal.pone.0225417 (PMC6897413; doi:10.1371/journal.pone.0225417)
Supplement: Attachment — Submitted filename: Response to Reviewers.docx [file pone.0225417.s005.docx]

**EDITOR’S COMMENTS**

1. Please ensure that your manuscript meets PLOS ONE's style requirements, including those for file naming. The PLOS ONE style templates can be found at

http://www.journals.plos.org/plosone/s/file?id=wjVg/PLOSOne_formatting_sample_main_body.pdf and http://www.journals.plos.org/plosone/s/file?id=ba62/PLOSOne_formatting_sample_title_authors_affiliations.pdf

**Response: Changes have been made to adhere to the journal’**

2. We note that you have indicated that Avertin was used to sacrifice animals in your study. We would be grateful if you could clarify how death was confirmed in these animals, following administration of Avertin in the dose described. If animals were decapitated or other secondary methods were used to ensure death please include this information in your Methods section.

Please also state whether your ethics committee specifically approved the use of this compound.

Please also provide the supplier of Avertin, or describe how it was synthesised.

**Response: Further details have been added in the manuscript in the methods section, lines 156-166.**

3. We also note that you have stated that "All animal experiments were carried out in accordance to the 86/609/EEC Directive (Cyprus Veterinary Services project license approving the experimental and sacrifice protocol CY/EXP/P.L6/2010).".

We would be grateful if you could clarify in your Ethics Statement and Methods section whether the study was approved by an ethics committee. If Cyprus Veterinary Services served as an ethics committee please include this information in your Methods section.

**Response: The protocol was approved by the Cyprus Veterinary Services, including the sacrifice method, number of animals euthanized and the method of retrieving tissues. This was clarified in the methods section as stated above.**

4. Thank you for stating the following in the Competing Interests section:

"The authors E.P. and T.K. have filed a PCT application on the use of the modified C5aR agonist EP67 in cerebral and peripheral amyloidoses (PCT/EP2018/053362)."

a. Please confirm that this does not alter your adherence to all PLOS ONE policies on sharing data and materials, by including the following statement: "This does not alter our adherence to  PLOS ONE policies on sharing data and materials.” (as detailed online in our guide for authors <http://journals.plos.org/plosone/s/competing-interests>).  If there are restrictions on sharing of data and/or materials, please state these. Please note that we cannot proceed with consideration of your article until this information has been declared.

b. Please include your updated Competing Interests statement in your cover letter; we will change the online submission form on your behalf.

Please know it is PLOS ONE policy for corresponding authors to declare, on behalf of all authors, all potential competing interests for the purposes of transparency. PLOS defines a competing interest as anything that interferes with, or could reasonably be perceived as interfering with, the full and objective presentation, peer review, editorial decision-making, or publication of research or non-research articles submitted to one of the journals. Competing interests can be financial or non-financial, professional, or personal. Competing interests can arise in relationship to an organization or another person. Please follow this link to our website for more details on competing interests: <http://journals.plos.org/plosone/s/competing-interests>

**Response: We confirm that the patent filing does not alter our adherence to all PLOS ONE policies, this was also included in the “Competing interests” section of the manuscript.**

5. Both reviewers raise some methodological concerns, as described in their comments.  I recommend to make an appropriate revision to address these criticisms carefully. Especially, it is necessary to consider seriously about the validity of some immunohistochemical analyses such as synaptophisin immunostaining. In addition, please provide the method of immunohistochemistry more in detail. Also please recheck the data of Fig 3c, in which very large reductions are observed in 5XFAD, compared with WT.

**Response: We have provided greater detail in the immunohistochemistry protocol and have reviewed our data and figures. New images of the immunohistochemistry experiments have been provided. We should also note that the quantification was only carried out through immunoblotting and immunoassays, while immunohistochemistry was only utilized as a means to confirm/visualize the trend.**

**REVIEWER #1 COMMENTS**

1. The agent used to treat the mice is a 9 amino acid peptide. They administer it orally in the drinking water for a week once each month. Most peptides do not survive transit through the GI tract. There is no demonstration that the drug gets into either plasma or the central nervous system. Do the authors have any pharmacokinetic data they can supply or at least refer to that demonstrates CNS penetration by this relatively hydrophilic peptide? Why did they not provide the agent continuously?

**Response: While we did not specifically investigate for the presence of the peptide in the brain and we could not find any existing literature pertaining to this action, this does not negate the net result. The significant increase of the CD88 receptor (C5aR1) in the brain (Supplementary Figure 2) demonstrates activation of the C5a-C5aR1 axis in the brain but does not prove that this happened locally that was not imported from the periphery. It is likely that both phenomena occur (Fig S3). We do have plans to investigate in depth the pharmacokinetic properties of the EP67 agonist and whether it can or not cross the blood brain barrier. EP67 was not administered continuously to avoid overstimulation of phagocytes and thus exacerbate neuroinflammation with a detrimental effect on amyloidogenesis.**

2. The authors claim they modified the peptide such that it activates the macrophage receptor, but not the neutrophil receptor. Are these two receptors different genes, or splice variants? How can this be achieved?

**Response: EP67 is a conformationally biased, response-selective analogue of the biologically active C-terminal region of human complement component C5a 65-74 (ISHKDMQLGR). The agonist was generated by substituting specific residues in C5a and attaching a methyl group to the nitrogen atom on the amide bond between proline and leucine. These structural modifications restrict and extend the backbone’s conformation creating biased topochemical features that allow for a conformational distinction between C5a-like inflammatory and immune stimulatory activity. These features therefore allow EP67 to interact with C5a receptors expressed on antigen presenting cells but is devoid of C5a-like neutrophil and neutropenic abilities (*Morgan et al., “Enhancement of in vivo and in vitro immune functions by a conformationally biased, response-selective aagonist of human C5a: Implications for a novel adjuvant in vaccine design.” 2009*)..**

3. The sample size is specified as 6 per group. They state that one hemisphere was fixed for histological processing and the other frozen. They then indicate that the Aβ measurement was performed on the entire hemisphere (line 186). The kits they used specify the use of guanidinium buffer, which would dissolve both fibrillar and soluble Aβ, yet they claim to only measure non-fibrillar Aβ. Was there a centrifugation step?

**Response: Since samples were to be used for western blotting we first pulverized the brain hemisphere with a medical scalpel and homogenized and sonicated the tissue further with PBS supplemented with the protease inhibitor cocktail (approximately 50μL) which resulted in a thick paste consistency. We then used the 100mg of this tissue preparation to carry out sample preparation for ELISA with the guanidine-HCL buffer as stated in the manufacturer’s protocol. The same tissue was also used for western blotting where the proteins were then extracted with RIPA buffer. This has been clarified in the materials and methods section (Lines 170-174). Below is a schematic of which tissue sections were used for which method. Regarding the species measured through the Elisa kits used we had previously contacted the manufacturing company with the same question and were told that the kit can only detect the soluble monomers due to the recognition site of the detection antibody since the C-terminal epitope would not be exposed in aggregated Aβ. At any rate this is a comparative study between treated and untreated 5XFAD animals.**

4. The authors then indicate that they used a RIPA buffer (line 199) to perform immunoblots. Where did the other tissue come from (unless they did not follow the Aβ kit instructions). They further indicate extracting total RNA (line 241). Where did the tissue for this come from? There needs to be some further methodological detail to indicate how they measured these multiple markers from the same tissue. Or perhaps, they did not make the measurements on the same tissue but had either dissected brain regions, more mice, or perhaps reduced the numbers of mice for each measurement.

**Response: Sections from the paraffin embedded 4% fixed hemisphere were used for RT-QPCR. This has also been specified in the methods section. Also please refer to lines 170-174, 178.**

5. The authors need to find a reasonable convention for specifying the Aβ peptide and stick with it. They variably refer to it as Amyloid β, as αβ, A4, or Aβ.

**Response: Aβ will be used throughout the manuscript, this has been amended accordingly.**

6. For immunohistochemistry, the authors need to specify the number of sections they analyzed for each stain and the distribution of the sections throughout the tissue. Paraffin sections are often sampling from a limited portion of the region and not very representative overall.

**Response: Immunohistochemistry was only carried out in order to visualize effects and was not used in a quantitative means, as this was provided by the immunoblotting, immunoassays and RT-QPCR. Fibrillar Aβ quantification with Thioflavin-S was the only immunohistochemistry quantified (please refer to). The conclusions of the current study are mainly based on immunoblots and ELISA while immunohistochemistry sections were used to provide a visual confirmation of the findings. Lines 235-244.**

7. Is there evidence that C5a increases the expression of its receptor? Typically, agonists decrease the cognate receptor. As this is the only evidence the authors offer of CNS penetration, it would be more convincing if this was a well known phenomenon.

**Response: We did observe and record a significant increase of the CD88 receptor (C5aR1) in the brain (Fig 3) as stated earlier which indicates that EP67 does increase the expression of its receptor in the brain not necessarily on individual cells but due to the increased recruitment of cells bearing the receptors.**

8. Line 288 seems a bit odd. First, most anti-Aβ antibodies list the aa in the Aβ sequence. It would appear that the aa listed (672-714) are from the amyloid precursor protein sequence, not the Amyloid A4 sequence (which is the same as Aβ). Second, it is also unclear what is meant by A4 precursors

**Response: This statement has been rephrased, line 304.**

9. The reductions in NeuN staining in the 5xFAD line of 70-80% are beyond any reductions reported previously. These mice have a modest reduction in neuron number in layer V of the anterior cerebral cortex. It is important that the authors specify what regions they are imaging and the number of measurements made per mouse if these data are to be believed. Further the images in the pdf are so dark that this referee is not able to evaluate what is being stained.

**Response: The images shown were only used for illustration purposes and immunoblots were instead used for quantification, Fig 8 illustrates closer images of the NeuN staining, these have now been taken in greater resolution in order to enable closer observation. The region from which immunochemistry sections are taken are from the posterior cerebral cortex. The immunoblot data are based on homogenates from the whole hemisphere.**

10. Figure S1 and S10 should probably be in the manuscript (although this referee was not able to view them). The results presented in S10 at least are integral to the overall interpretation of the mechanism by which this agent appears to prevent amyloid induced changes in the mice.

**Response: Supporting figures have now been included in the manuscript.**

In summary, this is a manuscript which examines a novel agent for amyloid reducing effects in a mouse model of amyloid deposition (its not really FAD without tau pathology and brain atrophy). There are some methodological issues which need to be addressed. It also should be the case that evidence that the agents does gain access to both plasma and brain should be at least referenced. Given that we have close to 500 manipulations that reduce amyloid in APP mice, it is unclear how much of an advance this is. However, the reported effect sizes are substantial and the approach is relatively novel.

**REVIEWER #2 COMMENTS**

In this study, Panayiotou et al., authors demonstrated that the reduction of amyloid-β (Aβ) accumulation in the brains and improvement of cognitive impairment in a model mouse of AD (5XFAD) by the intermittent administration of a modified C5a receptor agonist EP67. Authors also suggested that this therapeutic effects of EP67 are exerted by the preservation of synaptic integrity following the promotion of Aβ clearance by microglia and infiltrated myeloid cells such as monocytes/macrophages but not astrocytes and neutrophils.

This manuscript suggests very important information and evidences for the development of a novel immunotherapeutic strategy against AD: the modulation of brain immunity through the regulation of compliment pathways, especially using a modified C5a receptor agonist, would be the attractive strategy for treating AD.

Text is well written, and discussions are quite fair and reasonable. However, the problem is the poor quality of immunohistochemistry. Except for figure 1a, immunohistochemical data including supplemental figures should be replaced to those of more good quality. Alternatively, it would be possible that authors delete the immunohistochemical analysis in this manuscript as the future study.
For example, staining of Synaptophysin and beta tublin III should be laminar but not dots nor like postsynaptic. In the NeuN staining, the nuclei of neurons are too big. GFAP and F4/80 signals should be detected over a wide area even in wild type mice, Iba1 and CD68 staining should indicate microglia but not neuron like structure. DAPI staining in figure S10 also shows too big nuclei.

Minor comment;
Please define and unify the short form of amyloid-β to ‘αβ’ or ‘Aβ’.

**Response: The immunohistochemistry images have been deleted from the quantification figures and instead different images have been added. In figure S10 (now Fig 13) there was an error with the scale bar which has been rectified.**
