# Supplementary material for: C5aR agonist enhances phagocytosis of fibrillar and non-fibrillar Aβ amyloid and preserves memory in a mouse model of familial Alzheimer’s disease
Source: PLoS One. 2019 Dec 6;14(12):e0225417. doi: 10.1371/journal.pone.0225417 (PMC6897413; doi:10.1371/journal.pone.0225417)
Supplement: Attachment — Submitted filename: Response to Reviewers.docx [file pone.0225417.s006.docx]

Additional Editor Comments:

In the revised manuscript, the authors addressed most of the comments raised by the reviewers. However, their responses to several comments are not sufficient; especially it is necessary to clarify the following points.

1. Regarding the issue of whether EP67 can get into either the plasma or the brain, the authors need to add more explanations in the discussion part.

**Response: This has been added to the manuscript in the discussion section, lines 544-550.**

2. Regarding the comment No2 of Reviewer#1, the response should be added somewhere in the manuscript.

**Response: This information has been added, lines 101-110.**

3. Methods of immunoblotting (page 11, 211-216) are not described clearly. This part should be corrected in such a way that other researchers can reproduce the experiments.

**Response: More details were added in the manuscript, lines 214-226.**

4. The subtitles “Fibrillar Abeta quantification” and “Non-fibrillar Abeta quantification” are not appropriate. The former means the quantification of Thioflavin S-positive Abeta plaques and the latter means the quantification of GuHCl-soluble Abeta40 and Abeta42 by ELISA. As the expressions “Fibrillar Abeta quantification” and “Non-fibrillar Abeta quantification” can be misleading, these should be rephrased throughout in the manuscript.

**Response: The subtitles were changed accordingly, lines 175 and 195.**

5. Fig 6B: The immunoblotting data of NeuN appears unnatural. As NeuN is a nuclear protein and is not easily extracted by RIPA buffer, the data does not appear to reflect the actual situation in the brain. It is recommended to delete this data and to leave only the immunohistochemistry data.

**Response: This immunoblot was deleted and modifications were made, lines 373-375, 385-387 and Fig 6.**
