# Supplementary material for: C5aR agonist enhances phagocytosis of fibrillar and non-fibrillar Aβ amyloid and preserves memory in a mouse model of familial Alzheimer’s disease
Source: PLoS One. 2019 Dec 6;14(12):e0225417. doi: 10.1371/journal.pone.0225417 (PMC6897413; doi:10.1371/journal.pone.0225417)
Supplement: Attachment — Submitted filename: Response to Reviewers.docx [file pone.0225417.s007.docx]

Additional Editor Comments:

1. The responses to comment No3 are not complete.
**Response: Further details have been added to the immunoblotting protocol in the manuscript, lines 227-257.**

2. Please correct the following points.

- L189 “Fibrillar Abeta quantification”

**Response: This heading has been changed, line 191.**

- L195 “soluble Abeta quantification” This means GuHCl-soluble.

**Response: This has been changed lines 197 and 198.**

- L323 “non-fibrillar peptides”

**Response: This has been changed line 343.**

- L197-198 “manufacturer’s instructions” This part should be described more in details.

**Response: Further details have been added to the immunoassay protocol in the manuscript, lines 199-208.**

- L373-375 “neuronal loss”
  Synaptophysin loss indicates synaptic loss, not neuronal loss.
  This part should be changed to “synaptic and neuronal loss”.

**Response: This has been changed lines 394 and 395.**
